# The Amalgam Question

**Published:** 1877-02

**Authors:** 


AMERICAN JOURNAL

OF

DENTAL SCIENCE.

Vol. X. THIRD SERIES—FEBRUARY, 1877. No. 10.

### ARTICLE I.

### *The Amalgam Question.*

BY PROF. HODGKIN.

Probably no more vexed question than the usefulness of amalgam for fillings has engaged the attention of the dental profession, and on no question in the range of our specialty has more accrimonious discussion been indulged in, no one thing has been so sweepingly condemned or largely praised, and it is perhaps not injustice to say that in both cases of condemnation and praise, has less discrimination been made, and less pains taken to work scientific investigations of the facts at command. Science here as elsewhere, is the test to which we must on all such questions finally appeal, and with her impartial hand the mists that so befog the truth here will one day be blown away and we will be able to know what in this day of large empiricism we so vaguely guess at. When a Hubert Spencer of dentistry shall arise, and generalize the facts worked up so far, and those that are likely to be worked up in the next few years by the industrious of the profession, it will be seen that the truth here has so many angles and facts, as to elude the measurement of short-sighted ordinary mortals. Doubtless some such Prophet will arise in dentistry, who with larger mind will grasp and generalize these isolated facts and until then it may not be amiss to put on nerved and group together from time to time the isolated experiments and conclusions of those who have of late so diligently and earnestly worked at this problem and perhaps deduct from them what may be of conclusion. It is well to remark in the outset that this subject though old, comparatively speaking, is so crude in so far as its scientific aspect is concerned as to be almost entirely unscientific, and that the little we so far know is so imperfectly known, and the bulk of the unknown so large when compared with the known as to make it almost a new study. Certainly the results of the experiments so far, are widely at variance with each other, different experiments by the same experimenters, yielding contradictory results, and similar experiments at the hands of different manipulators differing fundamentally and widely. Research so far has hardly begun, and it is a most inviting field for the earnest investigator. One fact strongly detracts from the mists of some of these investigators, that is, that they, the experimenters are manufacturers, and so not likely to render unbiassed judgment. Still, their investigations are not without merit, and time to throw no little light on this subject, on which, as the following extract from late journalistic literature show, no little obscurity rests.

Fletcher of England: A serious fault developed in a number of amalgams examined, was in their tendency to assume a sphiroidal form whilst soft, showing a disposition to round off sharp edges and corners, so destroying accuracy of adaptation. He considers this a more serious fault than moderate shrinkage. Experiments with the silver (2) tin (1) and gold (10) per cent. alloy, chemically pure metals, demonstrated the expansion nearly alike in all the samples tested. Fillings in gloss cups, covered with ink as soon as inserted, assumed the sphiroidal condition in twenty four hours, allowing penetration of the ink to the bottom of the cups. One filling as above, left dry for twenty four hours and then covered, was a moisture-tight fit. He thinks the "out packing" test a crucial one as regards amalgam, and claims that his Platinum Amalgam will stand this test. He further asserts that chemically pure metals are out of the question commercially, on account of their cost.

Analysis of an amalgam filling thirty years old, showed nearly two thirds mercury (one third silver coin) present, visible signs of expansion were present and no trace of decay; filling and tooth both much discolored.

Keiley, of England: All the samples of amalgam that were tried, (most of those in common use) as a rule contracted; in all cases contraction, or expansion as the case might be, went in for hours or days after [apparent] complete hardening. Pure filed silver expanded for several days .025 inches, most amalgams containing more silver than tin expanded, but a large excess of this metal silver caused brittleness and discoloration; the best results seemed to be when the quantities of silver and tin bou relation to their atomic weights two atoms S. to one T. and 7 per cent. gold, a little gold rendering it more plastic, and hastening the setting. The variation in bulk of these amalgams (containing an excess of silver) is capricious, some contracting first and expanding afterwards, and others reversing this molecular movement, others again first expand, then contract, then expand again.

In practice, the dryer the amalgam the greater the warping, and all the amalgams worked dry, (*i. e.*, with little mercury;) there was a change in shape caused by the pres ure on the surface drawing the mercury to that part, and a subsequent absorbtion of this fluid by the alloy, causing expansion of the lower and contraction of the upper part of the filling.

Some shrinking amalgams fell out of glass tubes in a few days; expanding ones projected from like tubes after a few days and on being ground off, projected again the next day.

The "test by ink" is unreliable; none of the brought fillings stood the test of an air pump.

Dr. H. S. Chase of St. Louis, found on testing six of the brands of amalgam in the market that all shrank, (leaked under the "ink test") except Fletcher's. The samples tested were immersed immediately on being filled, examined in twenty four hours, finding slight leakage (in those that leaked;) and again in seventyt-wo hours, the last examination showing increased leakage.

Dr. Southworth expressed the mercury from a newly mixed amalgam, filling until the quantity in 19 grains of the proportion (fillings) only 9 grains of mercury remained. He found this a dry powder, but capable of welding, and finished up hard in a few minutes. The same conclusion he says was arrived at by Fletcher.

Dr. S. B. Palmer thinks that the addition of different metals to one amalgam only complicates and increases galvanic action, which acting on the tooth (or filling) is disintegrating in effect.

Dr. Cutler thinks that, equal skill exerted, amalgam will in most cases outlast gold. His statements of its durability are sweeping, holding that if the teeth in one side of the mouth be filled with gold and the other side with amalgam, this last will preserve the teeth much more effectually.

Dr. Hitchcock thinks that amalgam should be malleted to prevent leakage, that it is as difficult to manipulate as gold; if carefully packed it will not warp.

Dr. Fitch thinks, too little time is spent in manipulating amalgam; he spends five or six minutes in rubbing it before introducing into the cavity, making a homogeneous mass.

Dr. Kingsley. With water and a piece of soap all or any mixed lot of amalgam can be washed away, and reduced to coloring matter. The washing is positively injurious.

Mr. Brownley, of England: It is impossible to subject any specimens of amalgam to satisfactory tests out of the mouth; in such experiments the chemistry of the question may fairly be excluded. Too much is expected in the way of solution of this subject by chemical explanations. If it be a chemical combination that takes place in this hardening of amalgam it is of the feeblest character.

Practically, so far as the mercury is concerned, there can be no perfect uniformity in an amalgam filling, *i. e.* uniform distribution of the mercury.

The settlement of the question of shrinkage must be refered to some other ground than that of the proportion of mercury present, as the tests applied failed to show that a less or greater proportion of mercury influenced the shrinkage. *All specimens examined shrank*, (the amalgam tested was Sullivan's.)

Fletcher's expanding amalgam, was tested by the same instruments and methods ; and a shrinkage even greater than that of the former demonstrated 1 in 125, or twice that of Sullivan's.

It was thought, from the behaviour of some of the tested pieces, that irregularities in the shape of a cavity might hinder or modify shrinkage, and the change occur interstitially —the rough walls of a cavity in a tooth might be availed of here to advantage. In such a case a mass more or less porous would be obtained, without loss of bulk- *i. e.* by decrease of density.

Very dry specimens exhibited spontaneous fracture ; hence a limit to the minimum amount of mercury is indicated.

It is thought that shrinkage sets in at once, and so an advantage might be gained by waiting as long as possible for this to occur outside the mouth ; amalgam passing through the first half its shrinkage in one-third the time as the latter half.

The amount of shrinkage likely to occur in an amalgam filling of ordinary size, would be not more than 1-2000 of an inch.

Dr. Bogue carefully tested forty eight samples, first for shrinkage, and afterwards in acidulated saliva ; teeth with amalgam fillings and also amalgam sample of the filling tested. Three months of chemical action on the fillings of teeth showed disintegration of the latter but not of the former, the aggregate weight of the amalgam fillings remaining substantially unchanged. The samples of saliva were carefully analyzed and no mercury in solution found in any of the forty eight.

But little variation was noticed in the shrinkage of the various samples that did shrink. Two expanded permanently—pure silver and palladium. All these fillings showed good margins after this these mouths, both in acidulated saliva, and were all tested in a solution of indigo for two days with uncertain results, some samples of the same sort leaking, whilst others did not. "Extra" amalgam stood the test fully as well, as did also Walker's, Holme's and Hood and Reynolds. The palladium did not leak, nor did Fletcher's. Dr. B. thinks it is unadvisable to wash amalgam, it being impossible to get rid afterwards of the water.

---

### ARTICLE II.

### *The American Academy of Dental Surgery.*

[Reported for the American Journal of Dental Science.]

